# Supplementary material for: Are carbon emissions trading and green financial instruments synergistic? -Comprehensive quantitative research based on content analysis
Source: PLoS One. 2024 Mar 7;19(3):e0298601. doi: 10.1371/journal.pone.0298601 (PMC10919601; doi:10.1371/journal.pone.0298601)
Supplement: S1 Data — (ZIP) [file pone.0298601.s001.zip › Data/地级市Z_F工作报告.zip!/╦─┤¿/╤┼░▓╩╨/└┤╫╘╣1⁄2╓┌║┼í╛┬φ┐╦╩2╛▌═°í┐_2017.docx]

各位代表：

我代表市人民政府，向大会报告工作，请各位代表审查，并请市政协委员提出意见。

过去五年工作回顾

过去五年，是雅安发展史上极不平凡的五年。面对“4·20”芦山强烈地震重创和宏观经济持续下行压力，在省委、省政府和市委的坚强领导下，在市人大、市政协的监督支持下，市人民政府团结和依靠全市人民，攻坚克难、顽强拼搏，抗震救灾和灾后恢复重建取得重大胜利，经济发展稳中向好，社会事业明显进步，人民生活显著改善，顺利完成了市三届人大一次会议确定的目标任务，全面小康建设迈出坚实步伐。

五年来，全力战胜灾难、奋力克难赶考，灾后重建取得重大胜利。“4·20”芦山强烈地震发生后，在党中央、国务院亲切关怀和省委、省政府坚强领导下，在社会各界大力支持下，雅安人民共克时艰、凝心聚力，打赢了抢险救援、过渡安置、灾后重建等一场场硬仗。在抗震救灾过程中，全市上下万众一心、众志成城，最大限度地挽救了群众生命，最大限度地降低了灾害损失。在灾后重建征程中，认真践行“中央统筹指导、地方作为主体、灾区群众广泛参与”的恢复重建新路子，按照省委“三年基本完成、五年整体跨越、七年同步小康”的重建目标，始终把灾后恢复重建作为雅安迄今最全面最系统的民生工程、最重大最现实的发展机遇和必须高标准高质量完成的重要政治任务，强抓不懈、克难奋进，截至2016年7月20日，2160个重建总规项目的完工率和投资完成率均超过99%，圆满实现“三年基本完成”目标，受到党中央、国务院和省委、省政府的充分肯定和高度评价。三年砥砺前行，坚持民生优先抓重建，民生事业迈上新台阶。震后12个月完成159396户农房维修加固，震后14个月完成88978户城房维修加固，震后20个月完成78792户农房重建，震后32个月完成33696户城房重建，625户特困群众、4482户建房困难群众分别于2014年春节、地震一周年入住新居，群众居住条件实现根本改善。如期完成雅安中学新校区等374所学校重建，全面完成市医疗服务中心等128个卫计项目重建，建成投用文化艺术中心、陆家坝体育中心等251个文体广新项目和一批社会保障、社会福利等项目，全市公共服务水平显著提升。坚持交通先行抓重建，基础设施实现新突破。国道108线、318线完成改造提升，新建国道351线，5条经济干线基本建成，农村公路、客运站点等项目全面完工，雨名快速通道、永兴大道、发展大道等建成投用，市域内交通更加便捷、对外通达能力全面提升。一批水利、能源、通信等基础设施建成投用，全市基础保障能力极大增强。坚持一体化发展抓重建，城乡建设展现新面貌。完成115个城镇体系建设项目和218个农村建设项目重建，城乡一体化发展格局基本形成。北外环线、大兴大桥、陇西河大桥建成通车，规划馆、科技馆、生态博物馆、张家山公园、北纬30度公园等建成投用，“一城两线四出口”改造全面推进，中心城区城市功能和形象极大提升。6个县城基础设施和公共服务设施更加完善，综合承载能力明显增强。一批风貌突出、特色鲜明、配套完善的新镇和新村先后建成，成为发展富民产业的重要载体。坚持长远发展抓重建，产业发展开创新局面。新建、扩建9个工业园区，引进中恒天汽车、王老吉等亿元以上产业项目149个，新的经济增长点正在加速形成;初步建成5个特色农业示范园和3条农旅融合发展特色产业经济走廊，成功创建首个全国休闲农业与乡村旅游示范市，一批重建新村成为融合发展示范点，重建带动效应逐渐显现，灾区“造血”功能不断增强。坚持生态保护抓重建，防灾能力得到新提升。冷木沟等235个重大地质灾害治理项目主体工程在2014年主汛期前完成并发挥功能，避险搬迁安置3330户，震后连续四年未发生因地质灾害引发的群死群伤事件。治理水土流失面积419万亩，恢复震损林地40万亩，修复大熊猫栖息地22万亩，有力筑牢了抵御自然灾害的安全屏障。

五年来，保持发展定力、坚定后发追赶，经济实力再上台阶。按照市委三届七次全会确立的“抓住灾后恢复重建大机遇，做好生态文明示范大文章，加快建设幸福美丽新家园”的总体思路，坚持在打重建“硬仗”中补发展“欠账”，坚定不移走后发追赶之路，全市经济实现了量的扩张和质的提升。经济规模不断扩大。全市地区生产总值连续跨过两个百亿元台阶、增长54.8%，全社会固定资产投资、社会消费品零售总额分别增长62.6%、82.7%。经济结构趋优向好。三次产业结构由16.3∶57.2∶26.5调整为14∶55.5∶30.5。民营经济增加值占地区生产总值比重提高3.7个百分点、达58.5%。发展效益持续提升。地方一般公共预算收入、人均地区生产总值分别增长44.9%、51.4%。城乡居民人均可支配收入分别增长64.6%、73.8%，增幅均超过全省平均水平。县域经济提速发展。县(区)地方一般公共预算收入均超亿元。县域地区生产总值、全社会固定资产投资分别增长59.4%、62.4%。天全、石棉、汉源、宝兴县被评为全省县域经济发展先进县，荥经县被评为全省民营经济先进县。

五年来，始终定向着力、突出特色发展，产业支撑不断增强。持之以恒调结构、促转型，特色产业发展壮大。新型工业主导地位凸显。四大骨干产业和六大特色产业加快发展，其中农产品加工产业和新材料产业产值超百亿元。工业总产值超10亿元企业达6户，规模以上工业企业新增115户、达330户。芦天宝飞地产业园区(雅安经开区)列入全省“51025”重点产业园区，汉源工业园区、石棉工业园区升级为省级工业园区，荥经工业集中区升级为省级循环经济示范区。特色农业稳步发展。“五雅”特色优势产业持续壮大，产业基地新增232万亩、达480万亩，“三品一标”农产品新增161个、达216个，市级以上龙头企业新增43户、达107户，农民专合组织新增1863家、达2531家。现代服务业快速发展。成功引进东方碧峰峡、万达广场、国盛商贸城等一批服务业重大项目，服务业投资增长131%。入选首批国家全域旅游示范区创建单位、国家级医养结合试点单位，纳入全省康养产业创新发展核心区;碧峰峡创建国家5A级旅游景区通过省级检查，国家4A级旅游景区达17个、数量居全省第二位，全市旅游综合收入年均增长20%。现代物流、现代金融、电子商务等服务业加快发展。

五年来，着眼固本强基、注重统筹谋划，基础条件跨越提升。大力推进城乡大提升和交通大会战，统筹加快水利、通信等基础设施建设，全市发展基础更加坚实。城乡建设扎实推进。城镇化率提高7.3个百分点、达43.8%。完成《雅安市城市总体规划(2013—2030)》和3个县城总体规划修编、100个镇(乡)和359个村庄规划编制，城乡规划体系更加完善。中心城区建成区面积增加13平方公里、达34平方公里，名山撤县设区，姚桥新城基本形成，大兴新区基础设施建设全面启动，老城区城市功能不断优化，雨城区、名山区、芦天宝飞地产业园区(雅安经开区)同城化步伐进一步加快。6个县城建成区面积累计达25平方公里，11个镇跻身全国重点镇，3个村入选全国首批“中国乡村旅游模范村”。省级文明城市创建通过验收，城乡环境综合治理深入推进。

基础设施更加完善。建成和在建高速公路376公里，改造提升国省干道879公里，新建和改建农村公路2283公里;雅西、雅乐高速公路建成通车，川藏铁路成雅段和雅康、峨汉高速公路建设稳步推进，雅安综合客运枢纽加快建设。永定桥水利工程、名山农村供水总厂等建成投运。大岗山、飞仙关、民治水电站投产发电，全市水电总装机容量达1267万千瓦。智慧城市加快建设，入选“宽带中国”示范城市。

五年来，坚持绿色发展、专注优势转化，生态红利持续释放。牢固树立“绿水青山就是金山银山”理念，把牢绿色发展方向，生态文明建设迈出重要步伐。生态优势巩固提升。“三大生态工程”持续实施，森林覆盖率达63.6%，稳居全省第一。城市建成区绿化覆盖率达42.6%，成功创建四川省园林城市。栗子坪自然保护区升级为国家级保护区，大相岭自然保护区升级为省级保护区。加快建设国家生态文化旅游融合发展试验区，入选首批国家生态文明先行示范区，被列为国家生态文明示范工程试点市，生态文明示范引领作用持续发挥。生态环境有效治理。大气环境质量不断改善，饮用水水源和良好水体得到有效保护。中心城区城市生活垃圾无害化处理率达99%，农村生活垃圾治理达到“五有”标准。累计淘汰76户企业落后产能，节能减排任务全面完成。强化环保执法，查处了一批破坏生态的违法行为。生态资源不断转化。成功创建首个全国绿色(有机)农业示范区，绿色(有机)认证面积达116万亩;蒙顶山茶、雅安藏茶等茶叶品牌效应不断扩大，茶叶综合产值达150亿元;建成省级以上生态旅游示范区3个，生态资源不断转化为产业优势和经济优势。

五年来，深化创新改革、扩大开放合作，发展活力竞相迸发。大力实施创新驱动发展战略，持续深化重点领域改革，不断拓展开放合作空间，发展动力进一步增强，发展活力进一步激发。创新驱动不断深化。攀西战略资源创新开发试验区加快建设，碲铋资源创新开发上升为国家战略。荣获国家科技奖7项，建成省级以上工程技术研究中心、科技企业孵化器6个，实施市级以上重大科技成果转化项目86个，高新技术企业达26户，高新技术产业产值增长75%。“互联网+”行动深入推进，大众创业、万众创新蓬勃开展。重点改革稳步推进。组建雅安农村商业银行，完成雅安市商业银行首轮增资扩股，引进2家商业银行在雅安设立分行，新增2家村镇银行。组建雅安城投公司、文旅公司和交建集团。成立西南联交所雅安分所、天府商品交易所汉源花椒交易中心。蒙顶山茶叶交易所成为全国茶叶商品定价中心。农村产权制度改革稳步推进，医药卫生、文化等体制改革继续深化，不动产登记职责机构整合、公务用车改革全面完成。开放合作持续深入。与对口援建市、周边市(州)和省直部门、国有大型企业开展战略合作，形成了全方位、多层次的开放合作发展新格局。成功承办第三届攀西战略资源创新开发试验区投资大会，高效参加西博会等重大投资促进活动，累计引进到位市外资金1455亿元。

五年来，突出以人为本、持续改善民生，群众福祉明显增进。把保障和改善民生作为工作的出发点和落脚点，办成了一批既利当前、又惠长远的民生实事。民生支出累计达674.6亿元，占一般公共预算支出的66%。脱贫攻坚成效明显。全面落实“六个精准”，大力实施“五个一批”，扎实推进九项重点扶贫工程，全市103768名农村贫困人口减贫85673人，贫困发生率从9.1%下降到1.6%。社会保障提质扩面。城镇新增就业10万人，城镇登记失业率控制在4%以内。社会保险制度实现全覆盖，城镇职工养老保险、城乡居民养老保险参保人数分别增长56%、71%，新农合参合率达99.7%。社会救助体系不断完善，在全省率先实现低保线和扶贫线“两线合一”，发放城乡低保、医疗救助、高龄补贴等救助和福利资金9.2亿元。改造危旧房棚户区3.5万套。社会事业协调推进。县域义务教育均衡发展全域通过国家认定，高考本科上线率逐年上升，雅职院成功创建省级示范高职院校。全市医院床位增加3628张、达11655张，医疗卫生服务体系不断完善。推出《大美雅安》《恩情永不忘》等一批文艺作品，公共文化设施实现免费开放。成功举办市第三届运动会，组织承办国家级、省级全民健身活动28次。切实做好妇女、儿童、老龄、残疾人、慈善、红十字等工作，防震减灾、统计、气象、档案、保密、科协、地方志、社会科学等工作扎实推进，外事、侨务和对台等工作取得新进展。

五年来，创新社会治理、致力共建和谐，社会大局保持稳定。“六五”普法全面完成，“法律八进”扎实开展，法治雅安建设取得新成效。在全省率先构建“政社互动”实体平台，市、县、乡三级群团组织社会服务中心体系建成并发挥积极作用。进一步完善立体化治安防控体系，依法严厉打击违法犯罪活动，人民群众安全感、满意度不断提升。建立社会稳定风险评估、矛盾纠纷多元化解等机制，一批突出问题和社会矛盾得到有效解决。严格落实安全生产责任制，切实加强重点行业和领域安全专项整治，安全生产形势总体可控。食品药品监管体系不断完善，群众“舌尖上的安全”得到有力保障。市级综合应急指挥平台建成投用，突发公共事件得到有效处置。国家安全、国防动员和民兵预备役工作进一步加强，民族、宗教工作有序推进。

五年来，坚持依法行政、加快职能转变，自身建设全面加强。深入开展党的群众路线教育实践活动、“三严三实”专题教育和“两学一做”学习教育，政府执行力、公信力和依法行政能力持续提升。主动接受市人大及其常委会的法律监督、工作监督和市政协的民主监督，645件人大代表建议意见、922件政协委员提案全部办结。全面取消非行政许可审批，精简行政审批事项175项，在全省率先推行“三证合一”登记制度，雅安成为国家工商总局委托地方设立的首个商标受理处试点城市。建立市政府常务会会前学法和法律顾问制度，完善政府工作规则，行政决策进一步规范。完成政务服务中心标准化建设，整合公共资源交易平台，政务服务体系不断完善。贯彻落实中央“八项规定”精神和省委省政府“十项规定”，大力整治“四风”，“三公”经费、政府文会数量持续下降。严格落实党风廉政建设责任制，查处了一批违纪违规案件。

各位代表，刚刚过去的2016年，是本届政府收官之年。一年来，我们以建设美丽雅安、生态强市为总揽，决战重建脱贫双攻坚，统筹推进政府各项工作，圆满完成了市三届人大六次会议确定的目标任务。预计全市实现地区生产总值540亿元、增长8%，地方一般公共预算收入32.18亿元、同口径增长10.8%，全社会固定资产投资550亿元、增长3.5%，规模以上工业增加值增长9.5%，社会消费品零售总额223亿元、增长11.5%，城镇居民人均可支配收入27343元、增长8%，农村居民人均可支配收入11164元、增长9.5%。着力推进灾后重建，截至2016年12月31日，2160个重建总规项目的完工率和投资完成率分别达99.86%、99.95%，灾后重建任务胜利完成。着力抓好脱贫攻坚，大力实施“五个一批”和九项重点扶贫工程，1.1万人减贫、45个贫困村退出的目标任务圆满完成。着力稳定经济增长，实施促进经济稳定增长和提质增效二十三条政策措施，切实抓好重大项目开工和建设，组织开展各类市场拓展活动，全面推进大众创业、万众创新，经济发展稳中有进、持续向好。着力调整产业结构，扎实推进供给侧结构性改革，淘汰11户企业落后产能，高新技术产业产值增长11%，第三产业比重提高0.8个百分点，发展动能明显增强。着力提升城乡面貌，中心城区“一城三廊六片”建设加快，城市管理五大专项治理行动成效明显;县城功能不断完善，7个“百镇建设行动”试点镇建设任务全面完成，建成幸福美丽新村200个。着力加快改革开放，以经济体制改革为重点的各项改革持续深入，区域合作不断拓展，为发展注入了新的动力和活力。着力改善人民生活，“十项民生工程”和“20件民生实事”全面完成，教育、卫生、文化、体育等事业协调推进，人民群众幸福指数持续提升。

五年艰辛奋斗，成绩来之不易。这是党中央、国务院和省委、省政府坚强领导、亲切关怀的结果，是市委统揽全局、科学决策的结果，是市人大、市政协加强监督、共同努力的结果，是对口援建市、省直部门和社会各界关心帮助、倾力支持的结果，是全市干部群众拼搏实干、艰苦奋斗的结果。在此，我谨代表市人民政府，向全市人民，向所有关心、支持和参与雅安建设发展的各界人士，表示衷心的感谢并致以崇高的敬意!

五年实践探索，启示弥足珍贵。我们深切体会到，做好政府工作：必须坚定政治定力、增强执行能力，把强化“四个意识”作为第一要求，坚决贯彻落实党中央、省委和市委的决策部署，始终做到思想上清醒、政治上坚定、行动上自觉;必须坚定后发追赶、转变发展方式，把加快发展振兴作为第一要务，适应把握引领经济发展新常态，大力提高发展质量和效益;必须立足市情实际、加快优势转化，把推进绿色发展作为第一路径，坚决守护绿水青山，让绿水青山变金山银山，走“绿而美、绿变金”的特色发展道路;必须全面解放思想、打破惯性思维，把加快改革开放作为第一动力，大胆创新创造，大力引资引智，不断激发发展动力和活力;必须坚持统筹兼顾、推动全域发展，把壮大县域经济作为第一支撑，鼓励县(区)因地制宜、彰显特色，实现错位发展、竞相发展;必须坚持发展为民、践行服务宗旨，把增进人民福祉作为第一责任，加大保障和改善民生力度，让全市人民共享发展成果。

在总结成绩的同时，我们也清醒地看到，发展不快、发展滞后仍然是雅安面临的最大问题。主要表现在：比较优势有待进一步转化，产业结构不尽合理，县域经济发展不足;开放合作整体水平较低，招大引强效果不明显;城镇化进程滞后，城乡发展不平衡;社会管理面临不少新情况，教育、医疗、养老等事业与群众的期盼还有差距;部分干部思想观念不够解放，政府自身建设有待进一步加强。这些困难和问题，我们将高度重视，切实加以解决，决不辜负人民的期待和重托。

今后五年主要目标任务

各位代表，“凡是过去，皆为序章”。今后五年，是我市发展振兴关键的五年，也是后发追赶任务艰巨的五年，更是大有作为、大有可为的五年。只要我们胸怀大局、顺应大势，百倍用心、千倍用力，就一定能实现追赶跨越、发展振兴。

综合分析雅安的发展形势，机遇与挑战并存，机遇大于挑战。机遇来自政策红利的不断释放。全国、全省经济长期向好的基本面没有变，国家推进供给侧结构性改革，实施新一轮西部大开发、全面创新改革试验等重大战略;省委、省政府研究制定推动芦山地震灾区发展振兴政策措施，为雅安加快后发追赶提供了历史机遇。机遇来自区位优势的不断凸显。雅安地处成都平原经济区，是链接攀西经济区、川西北生态经济区的关键节点，具有融入成都、辐射康藏、链接攀西、抱团乐眉的区位优势，为雅安加快后发追赶提供了广阔空间。机遇来自发展基础的不断夯实。灾后重建极大改善了雅安基础条件，重建综合效应正在显现，广大干部群众渴盼发展的愿望更加强烈，为雅安加快后发追赶奠定了坚实基础。同时，我们也看到，全省各地竞争日趋激烈，标兵提速、追兵逼近，给我们带来巨大的挑战。发展时不我待，使命催人奋进。我们必须担负起承前启后、继往开来的历史使命，心无旁骛抓发展，一门心思干事业，坚定不移推动经济社会跨越发展。

市第四次党代会确定了我市今后五年工作的奋斗目标和主要任务，这是做好政府工作的行动纲领。今后五年政府工作的总体要求是：高举中国特色社会主义伟大旗帜，以马克思列宁主义、毛泽东思想、邓小平理论、“三个代表”重要思想、科学发展观为指导，深入贯彻党的十八大和十八届三中、四中、五中、六中全会精神，深入学习贯彻习近平总书记系列重要讲话精神和党中央治国理政新理念新思想新战略，统筹推进“五位一体”总体布局，协调推进“四个全面”战略布局，认真落实省委“三大发展战略”，坚持稳中求进工作总基调，保持打“硬仗”、补“欠账”的精神状态，以后发追赶为主线，以脱贫攻坚、发展振兴为重要任务，坚持走“绿而美、绿变金”的特色发展道路，实施生态环境、产业发展、基础条件、城乡建设“四大提升工程”，突出项目、开放、园区、企业“四大工作抓手”，强化人才支撑，推进创新驱动，确保与全国全省同步全面建成小康社会，加快建设美丽雅安、生态强市。

今后五年的主要奋斗目标是：

——综合实力大幅提升。保持高于全省的经济增长速度，经济发展质量和效益得到明显提升，力争地区生产总值迈上千亿元台阶，地方一般公共预算收入突破50亿元，三次产业结构不断优化，区域竞争力明显增强。

——人民生活持续改善。2018年底前农村建档立卡贫困人口全部脱贫、贫困村全部退出，动态消除绝对贫困。力争城乡居民人均可支配收入均增长50%。公共服务和社会保障体系更加健全，人民群众获得感、幸福感明显增强。

——生态文明建设特色彰显。生态环境受到严格保护、生态优势得到有效转化、生态经济实现快速发展、生态红利持续富民强市、生态家园充分彰显魅力、生态文明能够全国示范，雅安的知名度、美誉度进一步提升。“绿变金”特色发展成效明显，建成全省新兴绿色经济增长极。

——城乡面貌显著改变。城乡规划、建设和管理水平大幅提升，一体化进程不断加快。城市布局更加合理，市政设施更加完善，市容市貌更加整洁，文化内涵更加丰富，宜居宜业宜游的现代化城市基本建成。

——发展环境不断优化。交通、能源等保障能力全面加强，政策体系不断健全，政务环境持续优化，投资洼地基本形成。重点领域和关键环节改革取得新进展，市场在资源配置中的决定性作用充分发挥。各项事业步入法治化轨道，人民群众法治意识、文明素养明显提高。

各位代表，要实现以上目标，在统筹推进、全面做好各项工作的同时，我们将重点抓好五个方面的工作。

一是坚持精准施策，打赢脱贫攻坚硬仗。按照“四年集中攻坚、两年巩固提升”总体安排和“人脱贫、村摘帽、创四好、同奔康”工作要求，把脱贫攻坚作为头等大事，扎实推进“六个精准”和“五个一批”，实施“3+13”政策组合，全力以赴、不胜不休，确保2018年全面完成减贫任务。着力拓宽群众增收渠道，深入开展“四好村”创建活动，持续巩固脱贫成效，确保与全国全省同步全面建成小康社会。

二是坚持绿色发展，推动经济跨越提升。按照“农业强底座、工业挑大梁、三产当尖兵”的产业发展定位，深入实施产业发展提升工程，不断构建绿色产业体系，切实增强综合实力。做强新型工业。坚定不移走工业强市之路，全市工业总产值突破2200亿元，培育年产值超100亿元企业2户以上、超50亿元企业5户以上，建成西南绿色生态(有机)农产品加工基地、全省重要的汽车及零配件生产基地、攀西战略性新材料基地，推动芦天宝飞地产业园区(雅安经开区)建成千亿元级园区、升级为国家级经济技术开发区。做优特色农业。以推进农业供给侧结构性改革为引领，突出安全、高效、绿色(有机)，大力发展“五雅”特色优势产业，不断提升“3+N”特色产业经济走廊和“1+8”农业园区建设水平，打造中国藏茶之乡，建成全国绿色茶产业基地、绿色果蔬基地和绿色道地中药材基地。做精现代服务业。大力推进生态文化旅游融合发展，打造中国国际特色旅游目的地，建成国家生态文化旅游融合发展试验区、国家全域旅游示范市。创新发展生态康养、现代物流、电子商务等服务业，打造川西医养健康城，建成进藏物资集散地、川滇藏战略物流中心和川西商贸服务中心。提升发展县域经济。按照“三大片区”定位，加快建设一批工业经济强县、现代农业强县和旅游经济强县，力争雨城区地区生产总值超200亿元，名山区和汉源、石棉、荥经、天全县地区生产总值均超100亿元，芦山、宝兴县地区生产总值均超50亿元。

三是坚持统筹推进，全面建设绿美城乡。深入实施城乡建设、基础条件、生态环境提升工程，建设幸福美丽家园，筑牢长远发展基础。推进城乡品质提升。加快建设川西枢纽和区域性中心城市、国际化旅游生态城市、四川省级历史文化名城，构建现代城镇空间结构体系，力争全市城镇化率达到50%。推动中心城区建设提速，力争人口达到50万、建成区面积达到50平方公里，建成姚桥现代生态宜居城区和大兴国家级生态示范城区，全方位加快雨城区、名山区、芦天宝飞地产业园区(雅安经开区)多组团、一体化发展。打造各具特色的县城，建设一批旅游、商贸等特色集镇。突出乡土韵味、体现乡愁记忆，打造一批“业兴、家富、人和、村美”的幸福美丽新村。建设川西交通枢纽。规划建设通用机场，加快“3+1+2”铁路网、“二环六通道三连线”高速公路网、“五纵七横”国省干线公路网等骨干交通网络建设，确保川藏铁路成雅段、成雅快速通道和雅康、峨汉高速公路建成投用，推进川藏铁路雅康段、雅眉乐铁路等项目建设。完善能源、水利、信息等基础设施网络。加快水电、风能、太阳能开发利用，打造西部清洁能源基地。完成铜头引水、九龙水库、南郊水厂引水等工程建设，切实增强供水保障和防洪减灾能力。推进互联网广泛深度应用，构建覆盖城乡、高效共享的现代信息网。厚植“绿而美”生态文明。加快国家主体功能区试点示范建设，建成国家生态文明先行示范区。全方位开展绿美雅安行动，建成省级森林城市，创建国家级风景园林城市。实行最严格的生态环境保护，强化环境污染整治。建设一批资源综合利用示范基地、循环经济园区，增强绿色产业发展支撑。

四是坚持创新改革，激发发展强大动力。以更加有力的措施推进创新改革和开放合作，加快形成蓬勃发展之势。强化创新驱动。积极参与成德绵全面创新改革试验，加快攀西战略资源创新开发试验区建设。建设国家级碲铋研发中心，建成一批国家工程技术研究中心。深化市校合作、政企合作，推动军民融合发展。实施创业雅安行动，推动大众创业、万众创新。全面深化改革。坚定不移推进供给侧结构性改革，不断深化国资国企、金融、农村等改革，让改革红利更多惠及人民群众。持续优化发展环境，增强企业家发展信心，促进实体经济持续健康发展。拓展对外开放。瞄准珠三角、长三角、京津冀、成渝西等重点区域，积极招大引强承接产业转移，力争引进一批世界五百强企业和国内百强企业，发展更高层次、更高水平的开放型经济。

五是坚持发展为民，增强人民群众获得感。坚持以人民为中心的发展思想，着力解决人民群众最关心最直接最现实的利益问题，让雅安的发展更有“温度”，让群众的幸福更有“质感”。持续加大民生投入，集中建成一批民生工程、办成一批民生实事。落实创业就业扶持政策，健全完善覆盖城乡的社会保障体系和救助体系。推动教育振兴发展，引进优质办学机构，建成一批省级示范高中，让孩子们在家门口就能享受到更优质的教育。提高医疗卫生服务水平，建设一批三级医院和国家、省级重点专科，打造川西医疗中心，创建国家健康城市，建成国家卫生城市。大力推动文化繁荣发展，协调发展体育、科技等社会事业。推进社会治理能力现代化，构建全民共建共享的社会治理格局。

2017年工作安排

各位代表，2017年是新一届政府工作的开局之年，也是实施“十三五”规划的重要一年。做好今年工作，意义十分重大。我们将认真贯彻落实党中央、国务院，省委、省政府和市委的决策部署，牢固树立和贯彻落实新发展理念，适应把握引领经济发展新常态，坚持稳中求进工作总基调，以建设美丽雅安、生态强市为总揽，全力做好政府各项工作，促进经济平稳健康发展和社会和谐稳定。

今年经济社会发展主要预期目标建议：地区生产总值增长8%以上;完成全社会固定资产投资560亿元，增长1.8%;规模以上工业增加值增长9%;社会消费品零售总额增长11%;地方一般公共预算收入增长7%;城镇居民人均可支配收入增长8%，农村居民人均可支配收入增长9%;城镇登记失业率控制在4%以内;城镇化率提高1.2个百分点以上;完成省定节能减排和环保目标任务。

为确保实现以上目标，我们将着力做好八个方面的工作。

一、打好脱贫攻坚战，为实现全面小康打下坚实基础

聚焦“两不愁、三保障”和“四个好”目标，强力推进“五个一批”和九项重点扶贫工程，实现9251人减贫、105个贫困村退出。

加快改善群众生产生活条件。统筹推进贫困村卫生室、文化室、通信网络和通村硬化路建设。全面完成162户农村危房改造，确保贫困群众住上安全住房。全面落实低保线和扶贫线“两线合一”，确保2598名低保兜底对象全部脱贫。加快实施教育扶贫工程，全面落实教育资助政策，阻断贫困代际传递。加快实施健康扶贫工程，全面落实医疗扶助政策，防止因病致贫、因病返贫。

加强产业扶持和就业帮助。用好用活贫困村产业扶持基金，全面实施贫困村“一村一品”“多村一品”产业推进行动，因地制宜发展特色种养殖业，引导具备条件的贫困村大力发展乡村旅游、农村电商等业态，带动贫困群众发展致富。继续加强就业培训和就业对接服务，积极开发公益性岗位，确保有劳动能力的贫困家庭至少有1人就业。

创新脱贫攻坚推进机制。完善脱贫攻坚督导工作常态机制，健全绩效考核问责制度。建立贫困动态跟踪机制，动态监测返贫情况。健全财政扶贫投入稳定增长机制，加大专项扶贫资金、涉农资金、社会组织非定向帮扶资金等整合力度。完善社会力量参与机制，引导更多社会资本参与脱贫攻坚。强化扶贫资金监督管理，确保阳光脱贫、廉洁脱贫。

二、大力推进项目建设，充分发挥投资拉动关键作用

突出项目工作在经济工作中提纲挈领的极端重要性，牢牢把握项目工作抓手，扎实开展“项目年”工作，拉动经济平稳较快发展。

强化项目储备申报。抢抓国家一系列重大政策机遇，主动对接国家重大投资工程包、重点专项规划和三年投资滚动计划，在产业发展、基础设施、社会民生等方面编制一批重大项目，确保储备项目投资动态保持在5000亿元以上。加强对上汇报衔接，积极争取中央和省预算内投资补助、国家专项建设基金、企业债券、产业发展基金等支持，确保更多项目进入国家和省上“盘子”。

着力加快项目建设。完善重大项目推进机制，着力强化对投资和重点项目工作的督查考核。开展项目建设环境集中整治，优化重大项目、重点企业政务服务，强化项目建设资金、土地、电力等保障。科学实施项目投资计划，全力推进重大项目开工和建设，实施市以上重大项目400个、完成投资310亿元，其中新开工项目110个、完成投资100亿元。

不断优化投资结构。在抓好基础设施和民生投资的同时，把扩大工业、服务业投资作为优化投资结构的重中之重，着力引进和实施一批产业性投资项目，确保完成工业投资120亿元以上、服务业投资350亿元以上。继续推出和实施一批政府与社会资本合作项目，鼓励民间资本进入基础设施、基础产业、公用事业等行业和领域。

三、大力实施产业发展提升工程，不断厚植发展优势

突出把握园区和企业工作抓手，加快产业转型升级，进一步提升产业发展水平。

加快新型工业发展。坚持走新型工业化道路，力促工业经济提质增效。大力推进工业园区发展。强化园区基础设施和公共服务平台建设，着力提升综合承载能力。推动雅双工业园、天全“园中园”、雨城“园中园”标准厂房建成投用，加快建设新能源汽车、新材料、生物医药等战略性新兴产业核心集聚区，推进芦天宝飞地产业园区(雅安经开区)创建国家级经济技术开发区工作。力争全市工业园区营业收入突破700亿元，其中芦天宝飞地产业园区(雅安经开区)营业收入突破300亿元。发展壮大骨干特色产业。全面实施《中国制造2025雅安行动方案》，推动制造业迈向中高端，力争四大骨干产业和六大特色产业产值分别达到560亿元、200亿元。加快中恒天汽车生产及品牌建设，推动配套项目落地;支持新筑通工新能源客车、物流车的推广应用，加快建安工业高端齿轮产业化等项目建设，延伸汽车(新能源汽车)产业链。加快一名微晶扩能、亿欣钙业二期等项目建设，抓好玄武岩纤维项目引进和建设，不断壮大新材料产业。加快三九医药产业园区建设，支持太时生物科技等发展壮大，延长生物医药产业链。全力支持企业做大做强。用好财政扶持、电力支持等政策措施，帮助企业解决发展中遇到的困难和问题。分类推进骨干龙头企业、优势成长型企业和中小企业发展，力争进入全省大企业大集团培育名单企业新增3户，工业总产值超10亿元企业新增2户、达到8户，规模以上工业企业新增20户以上。组织实施11个重点技改项目，完成技改投资50亿元以上。

加快现代农业发展。深化农业供给侧结构性改革，把增加绿色优质农产品供给放在突出位置，狠抓农业产业结构调整和品牌建设，促进农业增效、农民增收。加快优势特色产业发展。坚持农村一二三产业融合发展，培育休闲农业、体验农业等农旅融合新业态。在稳定粮食生产的同时，继续发展壮大“五雅”特色优势产业，提升特色产业经济走廊建设水平，新发展特色优势产业基地3.6万亩。支持蒙顶山茶业公司与川投集团开展战略合作，依托国家茶检中心、蒙顶山茶叶交易所等平台，不断提高雅茶的市场竞争力和产业效益。努力引进林竹精深加工龙头企业，加快竹活性炭等项目建设，促进雅竹产业加快发展。建设现代农业园区。持续推进雅安国家农业科技园区扩园强区工作，加快名山国家茶叶公园、芦山现代生态农业示范园等建设和发展，力争全市农业园区综合产值达到150亿元。强化农业标准化和农产品品牌建设。巩固提升国家绿色(有机)农业示范区建设成果，加快农产品质量安全溯源体系建设，切实做好农产品商标注册和品牌认证工作，培育壮大“雅字号”农产品品牌，新增绿色(有机)认证面积14万亩、“三品一标”农产品35个。培育新型农业经营主体。扶持龙头企业、专合组织、家庭农场等加快发展，新增市级以上龙头企业30户、省级示范家庭农场6家，规范发展农民专合组织100家，产业化龙头企业销售收入增长8%以上。

加快现代服务业发展。积极对接全省服务业“三百工程”，着力构建现代服务业体系，促进服务业质量提升、比重提高。大力发展生态文化旅游产业。推进国家生态文化旅游融合发展试验区建设，加快创建国家全域旅游示范市。推动碧峰峡创建成国家5A级旅游景区，加快推进蒙顶山创建国家5A级旅游景区工作，推动牛背山、龙苍沟、二郎山、王岗坪等一批旅游项目开发建设。加快大熊猫旅游核心区建设，打造大熊猫文化旅游走廊和大熊猫生态精品旅游线路。积极承办四川国际文化旅游节，精心举办第十三届蒙顶山茶文化旅游节等节庆活动，着力提高旅游营销实效，实现旅游综合收入增长18%以上。大力发展生态康养产业。推进国家级医养结合试点单位建设，加快九襄阳光康养生态旅游小镇、花间堂(蒙顶山)精品旅游度假小镇等项目建设;鼓励养老机构与医疗卫生机构开展合作，在7家二级以上综合医院开设老年病科，新增民办养老床位700张。大力发展现代物流业。开工建设中国供销川西农副产品物流园区，加快雅安物流园区基础设施、川滇藏商贸物流园区汽配市场建设，不断完善“一园区、三中心、四服务站”现代物流体系。大力发展现代商贸服务业。建成万达广场、国盛商贸城二期等城市商业综合体，积极引进国内外高端品牌和知名商贸流通企业入驻雅安。

组织参加“惠民购物全川行动”“川货全国行”“万企出国门”等活动，不断释放消费潜力。统筹加快现代金融、电子商务等服务业发展。

四、大力实施城乡建设提升工程，加快推进新型城镇化

按照中央“五大统筹”要求，加快建设布局合理、功能完善、环境优美、绿色低碳、和谐宜居的现代城乡。

着力完善城乡规划。强化规划引领，逐步建立覆盖全域的城乡规划体系。完成《雅安市城市总体规划(2013—2030)》实施评估、名山区控制性详细规划报批及备案，加快历史文化名城保护、城市总体风貌综合提升、地下空间综合利用等规划编制。完成天全、芦山、宝兴县城总体规划修编，加快镇(乡)、村庄规划编制。

全力加快城乡建设。持续推进中心城区建设。以“一城三廊六片”建设为重点，不断完善中心城区功能。加快大兴新区建设，大力推进南外环线、滨江生态绿廊、大兴核心区生态工程等项目建设，确保桃花岛湿地公园建成投用。完善姚桥新城配套设施，启动高颐阙民俗文化风情街区建设，加快雅安综合客运枢纽工程建设。提升老城区城市品质，加快周公河大桥、女娲广场等项目建设。加快名山区城市建设，启动公交客运枢纽工程建设，推进名山河生态修复和景观提升，实施城市出入口形象提升工程。启动雨名连接线名山段建设，加快草坝至大兴连接线建设，大力推进雅安市生活垃圾焚烧发电项目建设，促进雨城区、名山区、芦天宝飞地产业园区(雅安经开区)同城化发展。统筹加快县城、重点城镇和幸福美丽新村建设。加快县城基础设施和市政公用设施建设，不断提升承载能力。支持九襄、多营等重点镇加快建设，促进农村居民就地就近城镇化。按照“小规模、组团式、微田园、生态化”的要求，建成幸福美丽新村300个。

大力加强城乡管理。坚持市县联动、全民参与，巩固省级文明城市创建成果，积极争创国家卫生城市。稳步推进城市管理体制改革，健全城市管理五大专项治理行动长效机制，促进城市管理工作规范化、制度化、常态化。加大市政基础设施建设力度，完成姚桥、城后路垃圾压缩中转站建设，新建立体停车位392个，建设便民出行充电桩40个，投放公共租赁自行车600辆，加强道路、园林、路灯、地下管网等设施管护。强化城镇生活垃圾和污水治理，扎实推进农村人居环境整治，不断改善城乡生产生活条件。

五、大力实施基础条件提升工程，持续改善发展条件

抢抓国家和省布局重大基础设施建设的机遇，着力完善交通、能源、水利等基础设施，更好支撑经济社会发展。

加快川西交通枢纽建设。持续实施交通大会战，大力推进区域交通互联互通。加强对外通道建设。加快推进川藏铁路成雅段、成雅快速通道和雅康、峨汉高速公路建设，扎实做好川藏铁路雅康段、雅眉乐铁路、泸石高速公路、成雅高速公路改扩建等项目前期工作。推进市内路网建设。全力推进多营青衣江大桥、省道105线雅安至上里段建设，开工建设省道433线泗坪经三合至泸定段、省道217线石棉至甘洛段，力争启动国道549线石棉段建设，扎实做好国道318线名山及雨城过境段、省道308线荥经至天全段、省道431线名山百丈经雨城上里至芦山龙门段等项目前期工作。改善提升农村公路350公里，不断优化农村公路网络。

加强能源基础设施建设。做好老鹰岩水电站项目前期工作，加快锅浪跷、出居沟等水电站建设。力争启动500千伏雅安送出加强工程建设，确保110千伏川藏铁路雅安牵引站供电工程、110千伏名山永兴输变电工程等项目建成投运。大力实施农网改造升级，切实做好老旧小区电力设施改造、城市配电网建设等工作。

加强农业、水利、通信等基础设施建设。新建高标准农田5.33万亩，新建堤防10公里。加快铜头引水、九龙水库、永定桥大田支渠扩建等工程建设，基本完成南郊水厂引水工程建设。启动公共信息服务平台建设，实施城市光纤入户工程和“宽带乡村”试点。

六、大力实施生态环境提升工程，不断夯实生态本底

以建设国家生态文明先行示范区为契机，着力治理生态环境，加强生态系统建设，让雅安的天更蓝、地更绿、水更清。

打好污染防治战役。落实环境保护党政同责和一岗双责，坚决打好大气、水、土壤污染防治“三大战役”。狠抓大气污染防治，全面淘汰黄标车，建成雅安市机动车尾气检测监控平台，突出治理PM2.5和PM10，确保空气质量优良天数比例达到92.9%。狠抓水污染防治，全面实行河长制，大力开展青衣江、大渡河等重点江河流域水污染治理工作，严格保护饮用水源。狠抓土壤污染防治，持续推进农村面源污染治理，着力加强土壤重金属污染源防控。加大环保执法力度，严惩环境违法行为。

开展绿美雅安行动。统筹实施绿美雅安“七大行动”，确保森林覆盖率达到63.8%。推进天然林资源保护、新一轮退耕还林等重点生态工程建设，完成营造林41万亩。改造提升城市绿地，建设城市滨水绿廊24公里。打造一批承载旅游功能的“至美绿道”，完成道路绿化美化530公里。改善提升水利风景区，建设河流渠系库区绿色生态廊道30公里。创建“绿美新村”60个。治理水土流失面积15万亩。加强生物多样性保护，推进大熊猫国家公园雅安片区建设。

持续转化生态资源。依托良好的生态资源，静心持久地发展绿色经济、生态经济、循环经济，把得天独厚的资源优势加快转化为独具特色的发展优势。加大技术改革创新，推进资源循环高效利用，促进循环经济形成规模和特色。严格环境准入，严控高耗能、高污染和产能过剩行业。

七、全面推进创新改革，加快培育发展新动能

纵深实施创新驱动发展战略，深入推进重点领域改革，大力振兴实体经济，不断扩大开放合作，形成后发追赶的强大合力。

大力推进创新驱动发展。加快攀西战略资源创新开发试验区建设，主动参与成德绵全面创新改革试验。推进军民融合发展，支持建安、雅化、川西等企业军民融合项目建设。支持企业建立研发机构，切实发挥企业创新主体作用。实施高新技术企业和产业产值“双增长”计划，确保高新技术企业新增3户、高新技术产业产值突破90亿元。加快科技成果转化，实施市级以上重大科技成果转化项目18个。大力推进大众创业、万众创新，培育认定市级以上孵化载体4家;积极鼓励雅商、农民工返乡创业，实施大学生就业促进和创业引领计划。

深入推进重点领域改革。深化供给侧结构性改革，持续推进“三去一降一补”。深化国资国企改革，加快国有企业结构调整和重组，做强做优国有企业。深化金融领域改革，加快雅安农村商业银行、雅安市商业银行改革，推动具备条件的企业上市挂牌融资。深化农村各项改革，统筹推进农村各类产权确权登记颁证工作，完善农村产权流转交易平台，加快供销社综合改革。大力推进财税、投融资等体制改革，有序推进资源要素配置市场化改革。加快机关事业单位养老保险制度改革，持续推进医药卫生、文化、教育等领域改革。

不断提升开放合作水平。突出把握开放工作抓手，力促开放型经济加快发展。深化区域合作。精准对接国家西向、南向开放和扩大内陆开放的战略布局，主动融入“一带一路”、长江经济带和成渝经济区发展。持续深化与市(州)、省直部门、大型国有企业和高校、科研院所的合作，推动一批项目落地实施。强化招商引资。大力推进驻点招商，积极探索市场化招商，深入开展委托招商、产业链招商、“互联网+招商”。积极参加中外知名企业四川行、西部国际投资大会暨进出口展等省级重大招商活动，精心举办汽车、旅游、康养等专题投资促进活动，着力引进一批重大项目。完善招商引资工作目标考核激励机制，加强项目落地督查，提高项目履约率、开工率和资金到位率。引进到位市外资金370亿元以上。实现外贸进出口总额4500万美元。

着力加强县域经济发展。完善区域发展推进机制，全面落实扩权强县和支持县域经济发展的各项政策，支持县(区)按照“三大片区”定位，加快转化比较优势，实现特色发展、错位发展、竞相发展。构建“亲”“清”新型政商关系，支持行业协会、商会发挥作用，不折不扣落实各项政策措施，切实维护民营企业合法权益，增强企业家发展信心，促进民营经济大发展、大提升，力争民营经济增加值占地区生产总值比重达到59%。

八、持续保障改善民生，切实增进人民群众福祉

始终把改善民生作为最大责任，守住底线、突出重点、完善制度、促进和谐，用改善民生的新成效不断淬炼发展“成色”。

倾力办好民生实事。持续实施民生工程和民生实事，确保民生支出占一般公共预算支出的66%以上。加强人力资源供求信息对接服务，加大对灵活就业、新就业形态的支持力度，城镇新增就业1.6万人。健全社会保险制度，实施全民参保计划，整合城乡居民基本医疗保险制度。做好社会救助，提高城乡低保水平，发展慈善事业，深化残疾人“量服”工作。改造危旧房棚户区3608套。推进既有住宅电梯增设工作。解决9251人安全饮水问题。大力实施“菜篮子”工程，落实常年蔬菜生产基地，稳定主要农产品生产与供应，保障人民群众日常生活需要。大力争取国家和省上政策扶持，加快革命老区、民族地区发展。持续抓好水电库区后续建设和发展。

加快社会事业发展。优化城乡教育资源配置，加强教师队伍业务能力和师德师风建设;扩大公益性、普惠性学前教育覆盖面，加快义务教育学校标准化建设，提高普通高中教育质量，创新推动职业教育发展，着力办好特殊教育和继续教育;全力支持民办教育发展，大力引进优质办学机构。全面实施城市公立医院综合改革和分级诊疗制度，加快川西区域医疗服务中心建设。坚持计划生育基本国策，有序实施全面两孩政策。不断完善城乡文化服务设施，推进公共文化服务体系建设;繁荣文艺创作，加强文物保护利用。落实全民健身战略，建设城市社区“15分钟健身圈”，扩大农村体育健身工程覆盖率。全面发展妇女、儿童、老龄、红十字等事业，切实做好防震减灾、统计、气象、档案、保密、科协、地方志、社会科学、外事、侨务和对台等工作。

提高社会治理水平。开展“七五”普法，引导群众合理合法表达诉求，提高全民法治意识。健全基层依法治理体系，支持工会、共青团、妇联等群团组织参与社会治理。推进“智慧公共安全”系统建设，健全社会治安防控体系，严厉打击违法犯罪活动。依法规范信访秩序，全力化解信访积案。整合各类调解力量，提高化解矛盾纠纷的能力和水平。强化交通运输、建筑施工、消防火灾、企业生产等安全监管，坚决遏制重特大事故发生。强化食品药品检验检测体系建设，提升监管能力，确保食品药品安全。加强应急管理，提升应急处突能力。持续做好国家安全、民族、宗教、人民防空、国防动员和民兵预备役等工作。

加强政府自身建设

各位代表，我们深知，人民群众对政府工作寄予厚望。我们将切实加强自身建设，不断提升履职能力和水平，努力建设人民满意政府。

强化依法行政，建设法治政府。深入贯彻《法治政府建设实施纲要(2015—2020)》，以法治思维和法治方式推动发展。自觉接受党内监督，主动接受市人大及其常委会的法律监督、工作监督和市政协的民主监督，认真听取民主党派、工商联、无党派人士和人民团体的意见建议，开放接受人民群众和新闻舆论监督。完善重大决策合法性审查和政府法律顾问等制度，将依法依规、阳光透明、集体决策作为行政决策的核心要件。健全行政执法责任制，深化“两法衔接”，加强行政执法队伍建设，提高执法能力和水平。

强化简政放权，建设服务政府。纵深推进“放管服”改革，不断完善权力清单、责任清单，开展相对集中行政许可权试点。加快打造实体服务大厅和网上办事大厅一体化的政务服务平台，推动行政审批和公共服务事项全部进驻。开展“减证便民”行动，坚决砍掉各类无谓的证明和繁琐的手续。推动公共资源更多地向民生倾斜，将第三方可提供的事务性管理服务主动交给市场，不断提高政府公共服务水平。

强化责任担当，建设效能政府。坚持求真务实，倡导事业为上、责任为重、工作为先，把心思集中在想干事上、把本领体现在能干事上、把措施落实到干成事上。加强干部教育培训，深化争创人民满意公务员活动，打造实干担当的公务员队伍。深化政府目标绩效管理，全面落实首问责任制、限时办结制和责任追究制，坚决整肃庸政、懒政、怠政行为，不断提升行政效能。

强化从严治政，建设廉洁政府。切实加强政府系统党的建设，把全面从严治党、从严治政各项规定落实到政府工作各环节。严格执行改进工作作风、密切联系群众相关规定，认真践行“三严三实”要求，驰而不息纠正“四风”。深化廉政风险防控，加强对行政权力运行的监督制约，扎紧制度笼子。充分发挥监察、审计等部门职能作用，坚持无禁区、全覆盖、零容忍惩治腐败。

各位代表，“积力之所举，则无不胜也;众智之所为，则无不成也”。让我们更加紧密地团结在以习近平同志为核心的党中央周围，在省委、省政府和市委的坚强领导下，紧紧依靠全市人民的智慧和力量，不忘初心，继续前进，为同步全面建成小康社会，加快建设美丽雅安、生态强市而努力奋斗!
